# Opposition to organ donation: prevention is better than applying tactics

**DOI:** 10.1186/s13054-014-0598-6

**Published:** 2014-11-06

**Authors:** Adriano Peris, Sara Bagatti, Paolo Lo Pane, Anna Nativi

**Affiliations:** Tuscany Transplantation Authority, Regione Toscana, (ITA) 26/N T. Alderotti St, 50139 Florence, Italy

One of the most important goals of a transplant system is the primary prevention of opposition to donation in order to maintain a balance between the objectives of transplant programs and respect for wishes regarding donation. Families play a major role in the consent to organ donation. The adoption of personalized organ donation directives (PODDs), as described in the editorial by Shaw [1] in a previous issue of *Critical Care*, offers a potential solution to the problem of family veto and may prevent the need to convince the family to donate at the time of bereavement. Shaw describes a complex system for recording advance directives apparently synergistic with a detailed protocol of persuasion in the case of opposition [2]. In some of the most demanding conditions - as in the case of organ donation from a no-heart-beating donor where there is little time for everything - behavioral and relational algorithms can easily degenerate into surrogates of actions that family members can find very invasive. Currently, the shortage of organs requires mainly a decisive intervention of information on the usefulness of organ transplantation, and this intervention cannot be replaced at the time of death by the work of persuasion, which is perceived as opportunistic and ethically questionable, or by a complex system of registration difficult to implement on a large scale.

## Author’s response

David Shaw

I thank Peris and colleagues for their thoughtful response to my article [1]. They argue that it is inappropriate to attempt to persuade families to permit donation after the patient has died. I disagree, but this is not the primary purpose of PODDs in any case.

Persuading families to permit donation can indeed upset them, but it is necessary for three reasons. First, in many cases, donation was the express wish of the patient and that wish should be respected. Second, most families who do not allow donation to proceed soon come to regret the decision to override their loved one’s last wish. Third, acquiescing with a family veto reduces the number of available organs, increasing the risk that other patients will die or suffer.

More importantly, however, PODDs are designed to prevent the need for post-mortem persuasion. If a donor creates a PODD and sends it to his or her relatives, they are much more likely to remember the wish and less likely to dispute whether the wishes are enduring because of the annual reaffirmation of donation intentions. PODDs are also useful for persuading families after a patient’s death if doing so is necessary, but in most cases it should not be. If Peris and colleagues wish to avoid distressing families, the PODD is a promising strategy for doing so, as it reduces the need for post-mortem persuasion.

## Abbreviation

PODD: personalized organ donation directive
